# Is Season of Diagnosis a Predictor of Cancer Survival? Results from the Zurich Cancer Registry

**DOI:** 10.3390/nu14204291

**Published:** 2022-10-14

**Authors:** Ola Hysaj, Nena Karavasiloglou, Manuela Limam, Miriam Wanner, Dimitri Korol, Sabine Rohrmann

**Affiliations:** 1 Division of Chronic Disease Epidemiology, Epidemiology, Biostatistics and Prevention Institute, University of Zurich, 8001 Zurich, Switzerland; 2 Cancer Registry Zurich, Zug, Schaffhausen, and Schwyz, Institute of Pathology and Molecular Pathology, University Hospital Zurich, 8091 Zurich, Switzerland

**Keywords:** season, cancer, survival, vitamin D, prognosis

## Abstract

In Switzerland, there is a large seasonal variation in sunlight, and vitamin D deficiency is relatively common during winter. The season of diagnosis may be linked to cancer survival via vitamin D status. Using data from the Cancer Registry of Zurich, Zug, Schaffhausen, and Schwyz with more than 171,000 cancer cases registered since 1980, we examined the association of the season of diagnosis with survival for cancers including prostate (ICD10 code C61; International Categorization of Diseases, version 10), breast (C50), colorectal (C18-21), lung (C34), melanoma (C43), and all sites combined. Cox proportional hazards regression models were used to assess the differences in the all-cause mortality by the season of the diagnosis. Winter was used as the reference season. Hazard ratios (HR) and 95% confidence intervals (CI) were calculated for all the cancers combined (excluding nonmelanoma skin cancer) and for prostate (in men), breast (in women), colorectal, lung cancer, and melanomas, separately. A diagnosis in summer and/or autumn was associated with improved survival in all the sites combined for both sexes (men: HR 0.97 [95% CI 0.96–0.99]; women: HR 0.97 [95% CI 0.94–0.99]) and in colorectal (HR 0.91 [95% CI 0.84–0.99]), melanoma (HR 0.81 [95% CI 0.65–1.00]), and breast cancer (HR 0.91 [95% CI 0.94–0.99]) in women. Our study results suggest that a cancer diagnosis in summer and/or autumn is associated with a better prognosis. The improved seasonal survival coincides with the seasonal variation of sun-induced vitamin D, and vitamin D may play a protective and beneficial role in cancer survival.

## 1. Introduction

There is mounting evidence in the recent years indicating that the season of diagnosis and the start of the treatment might affect the prognosis of several cancers such as colorectal [1,2], prostate [2,3], lung [1,4,5,6,7], breast [2,7,8], and even melanoma [9,10].

The Thames Cancer Registry reported that the long-term survival (>5 years) was statistically significantly better among lung cancer patients diagnosed in autumn compared with the cases diagnosed in winter [1]. Another study, conducted in Norway, reported similar findings, wherein the cases diagnosed in autumn had a better survival than cases diagnosed in winter [6], but the results were inconsistent for breast and prostate cancer. In the Norwegian cancer registry studies, both breast and prostate cancer cases that were diagnosed in summer and autumn had better survivals [3,8]. In the Thames Cancer Registry, this effect was observed for breast but not prostate cancer [1,7]. A study conducted in Sweden observed effects that were opposite to most of the previous results and reported a higher mortality in men and women diagnosed with cancer in summer [11]. One of the first studies that examined the question of seasonal effects also reported a worse prognosis of Finnish breast cancer cases diagnosed in September and October compared with cases diagnosed during the rest of the year [12]. For colorectal cancer, in the Thames Cancer Registry study, no statistically significant difference in the survival of patients was observed between summer/autumn and winter after the first month of diagnosis [1]. In contrast, consistent evidence in Norway reported that both men and women exhibited better survivals when diagnosed in autumn compared with a diagnosis in winter [2,6,13]. The association between the season of cancer diagnosis and survival for all the cancers combined was examined in a Thames Cancer Registry cohort. The men and women diagnosed in summer and autumn had a lower mortality risk than those diagnosed in winter or spring [7,8].

This epidemiological evidence could indicate that the seasonal variation of sunlight exposure might have a beneficial influence on cancer survival via its role in the cutaneous production of vitamin D. Vitamin D is endogenously produced in the skin following exposure to the ultraviolet B (UVB) wavelength in sunlight, contributing to almost 90% of the serum vitamin D levels; only a small amount derives from the diet and dietary supplements [14,15]. Although the cancer-protective mechanism that vitamin D has is not clearly understood, in vitro studies support the hypothesis that vitamin D derivatives have anti-proliferative effects and propose a plausible biological explanation for the observed seasonality of cancer survival [16,17]. In countries located in high latitudes (from 40° North to 64° North) including Switzerland (46° North), the season is the main factor influencing serum vitamin D levels, with the minimum levels between the beginning of November and the end of February [18,19]. Furthermore, in a recent study in Switzerland, vitamin D insufficiency during winter reached almost 60% in the general adult population [20].

As far as we know, the association between the season of a cancer diagnosis and the survival of cancer patients in a middle-latitude (or central) European country has not been examined. We aimed to investigate the possible seasonal fluctuations in cancer survival in Switzerland using the Cancer Registry of the cantons of Zurich, Zug, Schaffhausen, and Schwyz, which is the largest Swiss cancer registry.

## 2. Material and Methods

### 2.1. Data

The Cancer Registry of the cantons of Zurich, Zug, Schaffhausen, and Schwyz is a population-based registry. In 1980, registration started in the canton of Zurich, while this occurred in 2011 in Zug, and in 2020 in Schaffhausen and Schwyz. The registry collects data on cancer incidence, treatment, and outcomes in permanent residents of these cantons. Only data from the canton of Zurich were used in our study to enable investigation of long-term trends. The routine indicators of data completeness and quality are good for the Cancer Registry. Only 2–3% of cancer cases were identified by death certificates (DCO cases) in the last couple of years. The percentage of histologically verified cases was at least 93% in recent years [21]. For the current study, all cases of primary malignant cancer diagnosed in the period from 1981–2017 were extracted from the registry database. In earlier years, no systematic follow-up was conducted at the Cancer Registry. Starting with incidence year 2003, a systematic 5-year follow-up was carried out (e.g., five years after a specific incidence year, all patients diagnosed in that year were followed-up actively). Since 2018, a yearly active follow-up is currently being conducted by matching data from the citizen service departments in the respective cantons with all the cancer patients. We excluded from the analysis cases with an unknown month of diagnosis, as well as DCO cases (no follow-up time). Our final analysis included 171,775 cases.

We calculated person–years at risk from the date of the initial cancer diagnosis to the latest date of follow-up or date of death, whichever came first. Season of diagnosis was defined using the date of diagnosis as follows: Winter (December–February), Spring (March–May), Summer (June–August), and Autumn (September–November). Age at diagnosis was stratified in five age groups (based on age at diagnosis, rounded down to the nearest whole number): <50, 50–59, 60–69, 70–79, and ≥80 years. Period of diagnosis was stratified in 10-year time intervals (based on the year of diagnosis): 1981–1989, 1990–1999, 2000–2010, and 2010–2017. To account for the socioeconomic status of the patients we used the Swiss neighborhood index of socioeconomic position (SEP) as a proxy [22]. SEP is associated with several health outcomes in the Swiss population, including cancer [22,23]. We determined SEP for each cancer patient matching on the community level.

Stages were defined as follows: Ⅰ, Ⅱ, Ⅲ, Ⅳ, and missing stage based on pathological and/or clinical Classification of Malignant Tumors (TNM; Tumor, Nodules, Metastasis). These definitions were performed separately for each localization according to the TNM classification of malignant tumors version 6 (incidence years up to 2009) and 7 (incidence year 2010 and onwards) [24,25]. Pathological TNM (pTNM) was used if available; otherwise, the clinical TNM (cTNM) was used instead. If cM was missing, it was assumed to be zero; if both clinical and pathological N/Ts were missing, both missing N and T were set to missing. For breast cancer, missing pN/cN were set to zero if cT/pT = 1. Type of treatment was categorized as follows: surgical procedure, chemotherapy, radiotherapy, hormone therapy, other, and missing type of treatment. We considered only the first primary treatments that were performed in the first 6 months.

### 2.2. Statistical Analyses

Cox proportional hazards regression models were used to assess differences in all-cause mortality in different seasons of diagnosis. All analyses were stratified by sex. Winter (providing the lowest vitamin D concentrations) was the reference season used by which other seasons were compared. The analyses were performed for four survival periods: 0–1, 0–5, and 0–10 years after diagnosis to study the seasonal effects in short-, medium-, and long-term survivals. To allow for comparison with previous studies we have included an analysis of variation in mortality with season of diagnosis that included all periods of follow-up taken as a total (up to 37 years). Hazard ratios (HR) and 95% confidence intervals (CI) were calculated for all cancers combined after adjusting for age at diagnosis, period of diagnosis, and SEP. Of these, prostate (C61, International Categorization of Diseases, version 10), breast (C50), colorectal (C18–C21), lung (C34), and melanoma (C43) cancer patients were also analyzed separately. Furthermore, we conducted a sensitivity analysis where we also adjusted for stage of cancer and type of treatment in prostate, breast, colorectal, and lung cancer using only cases diagnosed in the period from 2003–2017 because the recording of cancer stage was not reported systematically prior to 2003. In order to handle missing values for stage and type of treatment, we applied multiple imputation with chained equations (MICE) to impute the incomplete data, assuming data were missing at random [26]. We categorized the missing variables before performing multiple imputation. We used a multinomial logit model to impute the missing variable categories. Considering that the amount of missing information was approximately 6% for type of treatment and up to 18% for stage, we imputed twenty-five data sets that were created based on the complete variables. *p* values < 0.05 were considered statistically significant. Analyses were performed using Stata software version 16.

### 2.3. Ethics

In the Canton of Zurich, all cancer cases are registered with presumed consent and registered based on a decision from 1980 by the Zurich Government Council and the general registry approval from 1995 by the Federal Commission of Experts for professional secrecy in medical research. In this analysis, all data were used anonymously, and no approval was required from the Ethics Committee of the Canton of Zurich.

## 3. Results

This analysis included 171,775 incident cancers after applying exclusion criteria. Of these, 82,543 were diagnosed in women and 89,232 in men. During a total follow-up time of 1,188,742 person-years, 109,927 (64%) deaths were recorded. Table 1 presents the distribution of the absolute number of cases and deaths and the respective percentages for the available characteristics by each cancer type and all cancers combined. There was no considerable seasonal variation in the number of diagnosed cases in any of the cancer types apart from melanoma.

For melanoma, we noticed an increased number of cases diagnosed in summer in comparison with other seasons. The mean age at diagnosis was 65.4 years.

Table 2 presents the association between the season of diagnosis and the time since diagnosis for women. At 0–1 year after diagnosis, a significantly reduced mortality was observed in those diagnosed in autumn for breast cancer (HR 0.86 [95% CI 0.74–0.99]) and all sites combined (HR 0.94 [95% CI 0.91–0.98]) compared with winter (Table 2).

Similar patterns, but no statistically significant associations, were seen in lung cancer and melanoma. For colorectal cancer, a significantly reduced mortality was observed in women diagnosed in spring (HR 0.81 [95% CI 0.71–0.91]). At 0–5 years after diagnosis for women, a significantly lower mortality was observed in cases diagnosed in autumn for all the sites combined (HR 0.97 [95% CI 0.94–0.99]) and a borderline significant lower mortality was also observed for melanoma diagnosed in summer (HR 0.81 [95% CI 0.65–1.00]). The pattern remained similar as for 0–1 year after diagnosis for breast and lung cancer in women, with HRs consistently lower when diagnosed in autumn, but no statistically significant results were observed. The results for colorectal cancer also remained consistent with that observed at 0–1 year after diagnosis, with the lowest mortality observed when diagnosed in spring (HR 0.87 [95% CI 0.80–0.94]). However, at 0–5 years after diagnosis, we observed a less substantial yet significant lower mortality when diagnosed in autumn, too (HR 0.91 [95% CI 0.84–0.99]). In a longer period of follow-up of 0–10 years following diagnosis, women with breast cancer (HR 0.91 [95% CI 0.94–0.99]), melanoma (HR 0.80 [CI 0.64–0.96]), and the combination of all the sites (HR 0.97 [95% CI 0.94–0.99]) exhibited a statistically significant better survival when diagnosed in autumn. However, we noticed a shift in the seasonality pattern where significantly better survivals were observed in patients diagnosed in summer for melanoma (HR 0.80 [95% CI 0.64–0.96]) and all sites together (HR 0.80 [95% CI 0.64–0.96]). For colorectal cancer in women, the greatest statistically significant mortality reduction was seen for the cases diagnosed in spring (HR 0.88 [95% CI 0.81–0.94]). A slightly lower statistically significant reduction was observed in patients diagnosed in autumn (HR 0.92 [95% CI 0.85–0.99]). In the overall follow-up, the seasonality pattern remained the same in women for colorectal cancer, melanoma, and all sites together, but the results were attenuated for breast cancer.

To further investigate whether the type of treatment or stage of cancer might affect the seasonality patterns observed, we conducted a sensitivity analysis for women with respect to three cancer sites: breast, colorectal, and lung cancers. Further adjusting for the type of treatment and the stage in women attenuated the seasonal variation observed in breast cancer, but this was not seen in colorectal cancer (Supplementary Table S1). The colorectal cancer cases diagnosed in autumn remained significantly associated with a better survival in the 0–5 years (HR 0.85 [95% CI 0.73–0.98]) and 0–10 years (HR 0.85 [95% CI 0.74–0.98]) periods after diagnosis and the overall follow-up (HR 0.86 [95% CI 0.75–0.99]).

In men (Table 3, Supplementary Table S2), we did not observe any significant seasonality pattern in any of the cancer sites we investigated separately (colorectal, prostate, lung cancer, or melanoma). However, we noticed a consistently significant better survival in the cases diagnosed in summer compared with winter when analyzing all the cancer sites together for 0–1 year (HR 0.96 [95% CI 0.93–0.99]), 0–5 years (HR 0.96 [95% CI 0.94–0.99]), and 0–10 years (HR 0.97 [95% CI 0.96–0.99]) after diagnosis. Further adjusting for the type of treatment and stage in men did not impact the results.

## 4. Discussion

In the present study, we analyzed the effect of the season on cancer survivals in Switzerland. Our results support the hypothesis that the season of diagnosis is associated with survival in cancer patients. The prognoses for breast and colorectal cancer as well as melanoma in women, and in all sites combined in both sexes, was significantly better when patients were diagnosed in summer or/and autumn when compared to the cases diagnosed in winter. No statistically significant associations between season of diagnosis and cancer survival were observed for lung and prostate cance.

Our findings are in agreement with previous reports where improved survivals were observed for breast [2,7,8,27,28,29] and colorectal cancer [7,8,13,27], melanoma [9,10,30], and all sites combined [1,7] when the diagnosis took place during summer or/and autumn, which the exception of a Swedish study [11]. Given that sunlight/UV exposure contributes to almost 90% of serum vitamin D, it has been proposed that the seasonal variations in vitamin D levels might be the underlying cause of the observed seasonality in cancer survival [31]. Several potential mechanisms could explain the cancer-protective effects of vitamin D and the impacts on cancer prognoses have been proposed. Evidence from experimental, laboratory, and epidemiological studies supports the hypothesis that vitamin D exerts antiproliferative effects that induce growth arrest and apoptosis through the activation of vitamin D receptors (VDR) [32,33,34]. The tumor-suppressing and differentiation-inducing activities of VDR has been shown and generalized to many cancer cell lines [32]. This biological evidence could explain the better survival we observed in women for all sites combined when the patients were diagnosed in summer/autumn or in men diagnosed in summer when their vitamin D levels are the highest. Similar findings were reported for all the cancer sites combined in the Thames Cancer Registry study cohort in the UK [1,7]. In addition, 1-alpha-hydroxylase is now known to exist normally in tissues other than the kidneys, including the breast, colon, and prostate. This allows for the intracellular production of calcitriol (1,25(OH)_2_D), vitamin D’s hormonally active form, which can exert antiproliferative and anti-metastatic effects [35,36,37]. 

The increase in the VDR expression in the above tissues suggests that breast, colorectal, and prostate cancers may be particularly susceptible to vitamin D treatment [8]. In agreement with this biological evidence, we observed significantly better survival in female colorectal and breast cancer patients when diagnosed in autumn. However, for prostate cancer we did not observe a similar mortality reduction in autumn as expected. In general, for prostate cancer, the results do not support an inverse association between vitamin D concentration and prostate cancer risk. Studies have reported contradicting findings regarding a seasonality at diagnosis pattern in prostate cancer patients [1,7,11]. In contrast to colorectal and breast cancers, prostate cancer cells have been reported to lose the ability to hydroxylate 25(OH)D to 1,25(OH)_2_D, such that circulation becomes the main source of 1,25(OH)_2_D [32].

In addition to its cancer-protective effects, vitamin D acts synergistically with several antineoplastic drugs and chemotherapeutic agents, thereby expanding its beneficial effects during treatment [38,39,40]. Furthermore, supplementation of vitamin D promotes apoptotic cell death during radiotherapy. The improved survival observed for cancers diagnosed in summer/autumn could reflect the beneficial effects of high levels of vitamin D during the period of cancer treatment via amplifying treatment effects.

For colorectal cancer, in the initial model that was adjusted for the age at diagnosis, the period of diagnosis, and SEP we observed better survivals for both diagnoses in spring and autumn. However, the effect in spring disappeared after adjusting for the stage of cancer and type of treatment in the sensitivity analysis, suggesting that the observed effect could have been due to the stage distribution. On the other hand, after an adjustment for stage and type of treatment, the mortality reduction for cases diagnosed in autumn increased (see Supplementary Material).

We observed a better survival in breast cancer patients when diagnosed in autumn at 0–1 and 0–10 years after diagnosis; these results were eliminated in the sensitivity analysis when adjusting for the cancer stage and type of treatment.

Melanoma presents a notable case in our study findings, because it is to a large part caused by sun exposure [41,42]. Only few studies have investigated the seasonal influence in melanoma survival, which have produced conflicting results [30,43,44].

We observed a higher absolute number of melanoma cases diagnosed in the summer and autumn than in the spring and winter, possibly linked to the skin awareness campaigns and increased reports of skin self-examinations since more skin is exposed during summer. However, those patients who were diagnosed in summer and autumn exhibited significantly better survivals. An explanation for this observation could be related to the earlier diagnosis of melanoma in summer, as it is associated with increased incidence or increased sun exposure. Previous reports point to the greater examination of skin in summer but also to the stimulation of melanocyte proliferation caused by the exposure of the skin to UV radiation. This is also supported by the increased DNA synthesis and pathological changes in nevi that lead to an increased diagnosis of melanoma in summer [45,46]. The higher numbers of cases diagnosed in summer and autumn in our data seem to support this hypothesis. Two previous reports have concluded that an earlier diagnosis in summer might account for the observed increased melanoma survivals in summer/autumn after taking prognostic factors—such as age, sex, year of diagnosis, tumor thickness, and anatomical site—into account [30,44]. Unfortunately, we could not address this hypothesis since the stage and other histologic parameters (i.e., mitoses, solar elastosis, and Breslow thickness) for melanoma were not available in our data. However, our findings agree with the improved survival for melanoma patients diagnosed in summer and autumn after adjusting for the patients’ and tumors’ characteristics [9,10,43]. These studies concluded that the improved observed survival might have other possible explanations besides the early-stage diagnosis in summer or autumn, suggesting that sun exposure-synthesized vitamin D may inhibit melanoma progression [43,47]. Melanoma cell lines are responsive to the antiproliferative effect of vitamin D by expressing the vitamin D receptor [47]. Another plausible explanation could be that sun exposure increases DNA repair capacity and induces melanization, which ultimately leads to better outcomes and less aggressive melanomas [43]. Given the major potential public health consequences, further in-depth studies of sunlight and vitamin D’s impact on melanoma prognoses are necessary in order to interpret the association of UV radiation exposure with increased melanoma survival.

Even though previous studies have been consistent in reporting better survivals among lung cancer patients diagnosed in autumn [1,4,5,6], we did not observe any significant mortality reduction. These results might possibly reflect differences in the stage distribution within the summer group as compared to the other seasonal groups. The seasonal effect for any of the cancer types examined might also be due to factors other than sunlight and vitamin D such as variations in health expectations or health care access or a relatively higher diagnostic for shift in the general mortality. The authors of the Swedish study that observed increased mortality in breast and prostate cancer patients in summer compared with winter argued that their results might be due to the structure of the Sweidish health care system and the traditional vacation time from mid-June to mid-August [11].

It is worth noting that in our analysis, we observed a less significant seasonal survival variation in men, and only when all the cancers were combined did men present significantly reduced mortality when diagnosed in summer. Some previous studies also observed a much stronger seasonal variation in women than in men [7,8]. Vitamin D deficiency appears to be less common in men than in women in the winter season [48,49]. These observed differences remain an area that needs further investigation.

Given the retrospective nature of our study, we cannot draw any conclusions as to the biological mechanism involved. Furthermore, the season of diagnosis has been used as a proxy for vitamin D, but this convenient method does not account for individual behaviors that might affect vitamin D levels. In addition, there are other factors that affect vitamin D status that we were unable to account for during our analysis, such as diet [15], ethnicity [50], education [51], and supplement intake [14]. Last, but not least, information on circulating vitamin D concentration is not available in large cancer registry cohorts.

Our investigation has several methodological strengths. We examined the effect of the season of diagnosis on cancer survival in a large sample of 171,000 patients that were diagnosed during a follow-up time of almost 40 years, relying on good quality data from the Cancer Registry of Zurich, Zug, Schaffhausen, and Schwyz. Our analysis yielded results that are consistent with the existing literature.

## 5. Conclusions

We observed a significant variation in the prognoses of cancer patients by the season of the diagnosis such that diagnoses in the summer and autumn months were associated with improved survivals, especially in colorectal, melanoma, and breast cancer in women and in both sexes for all the sites combined. Our results support the growing evidence that vitamin D may play an important role in cancer survival. If vitamin D turns out to improve the survival of cancer patients, this might be an easy-to-implement measure in clinical practice.

## Figures and Tables

**Table 1:** Number of cases, deaths, and percentage distributions of available characteristics by cancer type.

	Breast				Colorectum				Lung				Prostate				Melanoma				All sites			
	Cases		Deaths		Cases		Deaths		Cases		Deaths		Cases		Deaths		Cases		Deaths		Cases		Deaths	
	n	%	n	%	n	%	n	%	n	%	n	%	n	%	n	%	n	%	n	%	n	%	n	%
Total n (death %)	26,318		12,417	47.2	18,723		12,905	68.9	17,122		15,549	90.8	23,702		12,914	54.5	10,076		3292	32.7	171,775		109,927	64.0
Age at diagnosis (years, %)																								
<50	5422	22.6	1283	10.3	1462	7.8	589	4.6	1167	6.8	958	6.2	3105	30.8	406	6.2	3105	30.8	406	12.3	26,323	15.3	8945	8.1
50–59	5726	22.0	2060	16.6	2627	14.0	1341	10.4	3245	19.0	2825	18.2	2638	11.1	668	5.2	1793	17.8	435	13.2	28,016	16.3	14,551	13.2
60–69	6282	24.0	2799	22.5	4678	25.0	2861	22.2	5449	31.8	4894	31.5	8353	35.2	3169	24.5	2050	20.4	714	21.7	44,179	25.7	26,981	24.5
70–79	5053	19.0	3126	25.2	5665	30.3	4361	33.8	5053	29.5	4736	30.5	8414	35.5	5485	42.5	1937	19.2	911	27.7	45,041	26.2	34,368	31.3
80+	3304	12.6	2664	21.5	4291	22.9	3753	29.1	2208	12.9	2136	13.7	4077	17.2	3542	27.4	1191	11.8	826	25.1	28,216	16.4	25,082	22.8
Year of diagnosis																								
1981–1989	5053	19.2	4170	33.6	4285	22.9	3849	29.8	4106	24.0	4045	26.0	3352	14.1	3211	24.9	1528	15.2	908	27.6	35,947	20.9	31,708	28.8
1990–1999	6135	23.3	3847	31.0	4488	24.0	3612	28.0	4132	24.1	4012	25.8	5254	22.2	4407	34.1	2098	20.8	943	28.7	40,261	23.4	31,146	28.3
2000–2009	7999	30.4	3254	26.2	5459	29.2	3613	28.0	4686	27.4	4352	28.0	8333	35.2	4038	31.3	2948	29.3	971	29.5	50,366	29.3	30,177	27.5
2010–2017	7131	27.1	1146	9.2	4491	24.0	1831	14.2	4198	24.5	3140	20.2	6763	28.5	1258	9.7	3502	34.8	470	14.3	45,201	26.3	16,896	15.4
Season of diagnosis																								
Winter	6401	24.3	3014	24.3	4610	24.6	3223	25.0	4211	24.6	3808	24.5	5912	24.9	3164	24.5	2232	22.2	746	22.7	41,681	24.3	26,657	24.3
Spring	6618	25.2	3135	25.3	4640	24.8	3176	24.6	4288	25.0	3918	25.2	6043	25.5	3356	26.0	2427	24.1	814	24.7	42,997	25.0	27,615	25.1
Summer	6560	24.9	3147	25.3	4830	25.8	3382	26.2	4379	25.6	3995	25.7	5731	24.2	3173	24.6	2887	28.7	941	28.6	43,918	25.6	28,421	25.9
Autumn	6739	25.6	3121	25.1	4643	24.8	3124	24.6	4244	24.8	3828	24.6	6016	25.4	3221	24.9	2530	25.1	791	24.0	43,179	25.1	27,234	24.8
SEP ^1^																								
Mean (SD)	66.7	(6.3)	66.8	(6.3)	66.6	(6.6)	66.6	(6.4)	66.0	(6.3)	66.0	(6.2)	66.8	(6.7)	66.8	(6.5)	66.6	(6.5)	67.0	(6.5)	66.6	(6.5)	66.5	(6.5)
Sex																								
Women	26,155		12,315	47.1	8882		6097	68.6	5343		4572	85.6	NA ^2^		NA ^2^		5156		1479	28.7	82,543		50,099	60.7
Men	NA ^3^		NA ^3^		9841		6808	69.2	11,779		10,977	93.2	23,702		12,914	54.5	4920		1813	36.8	89,232		59,828	67.1

^1^ SEP Swiss neighborhood index of socioeconomic position, range: 0 (lowest) to 100 (highest); ^2^ NA = not applicable; ^3^ Breast cancers were only investigated in women.

**Table 2:** Mortality Hazard Ratios by season of diagnosis and time since diagnosis in women.

Cancer Site	Season of Diagnosis	Time since Diagnosis			
		1 Year Hazard Ratio (95% CI) ^2^	5 Years Hazard Ratio (95% CI) ^2^	10 Years Hazard Ratio (95% CI) ^2^	Total Hazard Ratio (95% CI) ^2^
Breast	Winter	1	1	1	1
	Spring	0.93 (0.81–1.08)	0.99 (0.93–1.07)	0.98 (0.93–1.05)	1.00 (0.95–1.04)
	Summer	0.95 (0.82–1.10)	0.99 (0.92–1.07)	0.94 (0.92–1.04)	0.99 (0.95–1.05)
	Autumn	0.86 (0.74–0.99)	0.98 (0.94–1.05)	0.91 (0.94–0.99)	0.99 (0.94–1.04)
Colorectum	Winter	1	1	1	1
	Spring	0.81 (0.71–0.91)	0.87 (0.80–0.94)	0.88 (0.81–0.94)	0.89 (0.83–0.95)
	Summer	0.92 (0.82–1.02)	0.94 (0.87–1.02)	0.96 (0.89–1.03)	0.94 (0.87–1.01)
	Autumn	0.92 (0.82–1.04)	0.91 (0.84–0.99)	0.92 (0.85–0.99)	0.93 (0.86–0.99)
Lung	Winter	1	1	1	1
	Spring	1.00 (0.91–1.11)	1.03 (0.94–1.12)	1.02 (0.94–1.01)	1.02 (0.93–1.11)
	Summer	1.00 (0.93–1.11)	1.02 (0.94–1.12)	1.01 (0.93–1.01)	1.01 (0.93–1.09)
	Autumn	0.95 (0.86–1.05)	0.96 (0.89–1.05)	0.96 (0.88–1.04)	0.96 (0.88–1.04)
Melanoma	Winter	1	1	1	1
	Spring	0.96 (0.63–1.46)	1.02 (0.82–0.1.3)	0.91 (0.76–1.09)	0.92 (0.79–1.06)
	Summer	0.77 (0.51–1.18)	0.81 (0.65–1.00)	0.82 (0.70–0.98)	0.70 (0.95–0.93)
	Autumn	0.70 (0.45–1.09)	0.90 (0.73–0.12)	0.80 (0.67–0.96)	0.71 (0.91–0.97)
All sites ^1^	Winter	1	1	1	1
	Spring	0.99 (0.92–1.01)	0.97 (0.94–1.01)	0.98 (0.95–1.05)	0.98 (0.95–1.00)
	Summer	0.96 (0.95–1.02)	0.98 (0.92–1.01)	0.97 (0.93–0.99)	0.97 (0.96–1.00)
	Autumn	0.94 (0.91–0.98)	0.97 (0.94–0.99)	0.97 (0.94–0.99)	0.97 (0.95–0.99)

^1^ Excluding nonmelanoma skin cancer. ^2^ Adjusted for age and period of diagnosis and SEP.

**Table 3:** Mortality Hazard Ratios by season of diagnosis and time since diagnosis in men.

Cancer Site	Season of Diagnosis	Time since Diagnosis			
		1 Year Hazard Ratio (95% CI) ^2^	5 Years Hazard Ratio (95% CI) ^2^	10 Years Hazard Ratio (95% CI) ^2^	Total Hazard Ratio (95% CI) ^2^
Colorectum	Winter	1	1	1	1
	Spring	1.03 (0.92–1.16)	0.99 (0.92–1.07)	1.00 (0.92–1.07)	0.98 (0.92–1.05)
	Summer	1.02 (0.92–1.15)	0.97 (0.90–1.04)	0.99 (0.96–1.06)	0.97 (0.94–1.01)
	Autumn	1.01 (0.91–1.14)	1.01 (0.94–1.10)	1.01 (0.95–1.08)	0.99 (0.93–1.07)
Lung	Winter	1	1	1	1
	Spring	1.01 (0.95–1.07)	0.99 (0.96–1.07)	1.02 (0.96–1.07)	1.02 (0.97–1.07)
	Summer	0.99 (0.93–1.06)	0.96 (0.96–1.04)	1.03 (0.97–1.08)	1.03 (0.98–1.09)
	Autumn	1.00 (0.94–1.06)	1.00 (0.93–1.08)	0.98 (0.94–1.04)	0.99 (0.94–1.05)
Prostate	Winter	1	1	1	1
	Spring	1.12 (0.98–1.28)	1.05(0.98–1.12)	1.04 (0.99–1.10)	1.03 (0.98–1.08)
	Summer	1.02 (0.90–1.17)	1.03 (0.96–1.10)	1.06 (0.98–1.11)	1.02 (0.97–1.07)
	Autumn	1.03 (0.90–1.18)	0.98 (0.91–1.04)	0.98 (0.93–1.04)	0.99 (0.95–1.03)
Melanoma	Winter	1	1	1	1
	Spring	1.05 (0.74–1.49)	0.94 (0.79–1.13)	0.93 (0.80–1.08)	0.98 (0.85–1.11)
	Summer	1.13 (0.82–1.57)	0.94 (0.79–1.11)	0.91 (0.79–1.06)	0.94 (0.82–1.06)
	Autumn	1.07 (0.76–1.51)	0.97 (0.81–1.16)	0.97 (0.84–1.14)	0.99 (0.88–1.15)
All sites ^1^	Winter	1	1	1	1
	Spring	1.03 (0.99–1.06)	1.01 (0.99–1.04)	1.01 (0.99–1.04)	1.01 (0.99–1.04)
	Summer	0.96 (0.93–0.99)	0.96 (0.94–0.99)	0.97 (0.96–0.99)	0.96 (0.94–0.99)
	Autumn	1.02 (0.98–1.05)	1.00 (0.97–1.03)	0.99 (0.98–1.02)	0.99 (0.99–1.03)

^1^ Excluding nonmelanoma skin cancer. ^2^ Adjusted for age and period of diagnosis and SEP.

## Data Availability

The data presented in this study are available on request from the corresponding author. The data are not publicly available due to data protection reasons.

## Supplementary Materials

The following supporting information can be downloaded at: https://www.mdpi.com/article/10.3390/nu14204291/s1, Table S1: Mortality Hazard Ratios by season of diagnosis and time since diagnosis in women (sensitivity analysis after 2003); Table S2: Mortality Hazard Ratios by season of diagnosis and time since diagnosis in men (sensitivity analysis after 2003).
