# Supplementary material for: Is Season of Diagnosis a Predictor of Cancer Survival? Results from the Zurich Cancer Registry
Source: Nutrients. 2022 Oct 14;14(20):4291. doi: 10.3390/nu14204291 (PMC9608958; doi:10.3390/nu14204291)
Supplement: Supplementary file 1 [file nutrients-14-04291-s001.zip › nutrients-1959764-supplementary.pdf]

## Supplementary Table S1. Mortality Hazard Ratios by season of diagnosis and time since diagnosis in women (sensitivity analysis after 2003).

| *Cancer site* | *Season of diagnosis* | *Time since diagnosis* | | | |
|---|---|---|---|---|---|
| | | *1 year hazard ratio 2* | *5 years hazard ratio 2* | *10 years hazard ratio 2* | *Total hazard ratio 2* |
| ***Breast*** | ***Winter*** | 1 | 1 | 1 | 1 |
| | ***Spring*** | 0.99 (0.75-1.26) | 0.98 (0.84-1.09) | 1.01 (0.88-1.05) | 0.99 (0.86-1.06) |
| | ***Summer*** | 1.02 (0.81-1.35) | 1.06 (0.95-1.24) | 1.05 (0.96-1.20) | 1.02 (0.95-1.16) |
| | ***Autumn*** | 0.87 (0.66-1.14) | 0.99 (0.89-1.16) | 1.06 (0.94-1.18) | 1.03 (0.92-1.14) |
| ***Colorectum*** | ***Winter*** | 1 | 1 | 1 | 1 |
| | ***Spring*** | 0.91 (0.72-1.10) | 0.90 (0.78-1.05) | 0.90 (0.78-1.03) | 0.89 (0.78-1.02) |
| | ***Summer*** | 1.03 (0.84-1.26) | 0.98 (0.84-1.14) | 0.99 (0.88-1.17) | 1.00 (0.88-1.16) |
| | ***Autumn*** | 0.87 (0.71-1.08) | **0.85 (0.73-0.98)** | **0.85 (0.74-0.98)** | **0.86 (0.75-0.99)** |
| ***Lung*** | ***Winter*** | 1 | 1 | 1 | 1 |
| | ***Spring*** | 0.95 (0.80-1.08) | 0.97 (0.85-1.08) | 0.97 (0.84-1.07) | 0.98 (0.83-1.06) |
| | ***Summer*** | 0.98 (0.84-1.13) | 1.00 (0.89-1.13) | 0.98 (0.88-1.11) | 0.98 (0.87-1.11) |
| | ***Autumn*** | 0.95 (0.82-1.10) | 0.97 (0.86-1.11) | 0.97 (0.86-1.09) | 0.95 (0.85-1.08) |

***Hazard ratios which differ significantly from unity (p < 0.05) are shown in bold***

**1 Excluding nonmelanoma skin cancer. –2 Adjusted for age and period of diagnosis, SSEP, stage and type of treatment**

***Imputed analysis**

## Supplementary Table S2. Mortality Hazard Ratios by season of diagnosis and time since diagnosis in men (sensitivity analysis after 2003).

| *Cancer site* | *Season of diagnosis* | *Time since diagnosis* | | | |
|---|---|---|---|---|---|
| | | *1 year hazard ratio 2* | *5 years hazard ratio 2* | *10 years hazard ratio 2* | *Total hazard ratio 2* |
| ***Colorectum*** | ***Winter*** | 1 | 1 | 1 | 1 |
| | ***Spring*** | 0.97 (0.81-1.23) | 0.88 (0.77-1.04) | 0.91 (0.82-1.06) | 0.91 (0.82-1.06) |
| | ***Summer*** | 1.04 (0.86-1.29) | 0.93 (0.81-1.01) | 0.96 (0.87-1.12) | 0.96 (0.87-1.12) |
| | ***Autumn*** | 1.01 (0.86-1.29) | 0.96 (0.83-1.15) | 0.96 (0.88-1.13) | 0.93 (0.86-1.06) |
| ***Lung*** | ***Winter*** | 1 | 1 | 1 | 1 |
| | ***Spring*** | 0.97 (0.86-1.09) | 0.98 (0.89-1.08) | 0.97 (0.88-1.07) | 0.99 (0.98-1.08) |
| | ***Summer*** | 0.99 (0.89-1.12) | 0.98 (0.89-1.08) | 0.98 (0.89-1.09) | 0.98 (0.98-1.09) |
| | ***Autumn*** | 0.96 (0.86-1.09) | 0.96 (0.86-1.05) | 0.94 (0.86-1.04) | 0.95 (0.94-1.05) |
| ***Prostate*** | ***Winter*** | 1 | 1 | 1 | 1 |
| | ***Spring*** | 1.06 (0.83-1.35) | 0.96 (0.86-1.09) | 1.02 (0.93-1.13) | 1.03 (0.94-1.13) |
| | ***Summer*** | 1.12(0.87-1.42) | 0.95 (0.84-1.07) | 1.03 (0.95-1.16) | 1.02 (0.93-1.25) |
| | ***Autumn*** | 1.06 (0.83-1.35) | 0.94 (0.85-1.05) | 0.99 (0.91-1.19) | 0.99 (0.90-1.09) |

**Hazard ratios which differ significantly from unity (p < 0.05) are shown in bold**

**1 Excluding nonmelanoma skin cancer. –2 Adjusted for age and period of diagnosis, SSEP, stage and type of treatment**

***Imputed analysis**
